# Biomarkers and Clinical Evaluation in the Detection of Frailty

**DOI:** 10.3390/ijms26167888

**Published:** 2025-08-15

**Authors:** Catherine Devitt, Devon Patel, Rustin Mahboubi Ardakani, Shaji Poovathoor, Zhaosheng Jin, Daryn Moller

**Affiliations:** 1 Department of Anesthesiology, Stony Brook University Hospital, Health Sciences Center, Level 4, Room 060, Stony Brook, NY 11794, USAshaji.poovathoor@stonybrookmedicine.edu (S.P.); daryn.moller@stonybrookmedicine.edu (D.M.); 2 Renaissance School of Medicine, Stony Brook University, Stony Brook, NY 11794, USA

**Keywords:** biomarker, diagnosis, inflammation, frailty, metabolomics, oxidative stress, screening

## Abstract

Frailty is a complex biological process that is associated with adverse outcomes in community-dwelling and hospitalized patients. While clinical evaluation is the current gold standard for screening and diagnosis, such an approach is not without its limitations (such as personnel and resource requirement). In this review, we will discuss prospective biomarkers for frailty. Opportunistic and deliberate radiological testing could provide important information that complements clinical frailty evaluation. Novel biochemical panels may yield additional methods for frailty screening in the future. It is known that early frailty intervention could lead to better outcomes for patients. Integration of electronic medical records, laboratory and radiological results, as well as clinical informatics infrastructure could result in augmented clinical decision-making and more optimized healthcare resources utilization.

## 1. Introduction

As the population ages, frailty has become a global healthcare and social care challenge. Frailty represents a loss of functional reserve and acquired vulnerabilities to stressors. Patients with frailty are at significantly increased risk of morbidity, mortality, and loss of functional independence, particularly after surgery or hospital admission.

Frailty is primarily diagnosed through clinical evaluation; however, the adoption of frailty assessment can be limited by provider and resource constraints. Understanding of the biological processes associated with frailty could lead to the development of biochemical or radiological tests for frailty screening. In the age of medical informatics, such tests could facilitate earlier frailty interventions and more efficient utilization of healthcare resources.

This review article will evaluate the utility of biochemical markers and radiological investigations in complementing the existing healthcare infrastructure for frailty screening and therefore create opportunities for earlier intervention. The authors searched the databases PubMed, Embase, and Google Scholar for relevant articles to this review. Article types considered for screening included review articles, basic science reports, clinical trials, observational studies, reports/series, meta-analyses, and systematic reviews. The references were screened by authors and selected for inclusion based on relevance to the topic and quality of evidence. The resulting body of evidence was synthesized by authors into this narrative review article.

## 2. Epidemiology of Frailty

Frailty represents a multifactorial state characterized by reduced physiological reserve and elevated vulnerability to stresses [1]. While physiologically distinct from aging, Searle et al. reported that the median age of frailty onset was 69 in a large European population [2]. According to the 2020 Census, the proportion of U.S. adults aged 65 and older makes up 16.8% of the total population [3]. Patients aged >65 also now comprised 30–40% of all surgical procedures [4,5]. The rapidly aging population and frailty bring with it a myriad of challenges to the delivery of hospital and community-based healthcare.

Frailty prevalence at the population level among adults differs based on age, sex, and the criteria used to define frailty [6]. Based on a physical deficit model, the highest prevalence of physical frailty was in Africa (22%) and the Americas (17%), while the lowest prevalence was in Europe at 8% [6]. In studies utilizing a frailty index, frailty was greatest in Oceania (31%), Asia (25%), the Americas (23%), and lastly Europe (22%) [6]. Based on both criteria, the prevalence of frailty is higher in females than males.

### Healthcare Outcomes

Surgical procedures are inherently associated with major physiological and psychosocial stress. Frailty has been strongly associated with elevated peri-operative risks of morbidity, mortality, neurocognitive decline, and impaired functional recovery [7]. Frailty was identified as an independent risk factor for mortality at both 30 and 180 days across all surgical specialties [8]. In the American College of Surgeons National Surgical Quality Improvement Program (NSQIP), very frail patients had a 30-day mortality rate of 13–15%, regardless of the surgery complexity [8]. The Veterans Affairs Surgical Quality Improvement Program (VASQIP) reported that frailty is an independent risk factor for postoperative mortality across surgical specialties and surgery intensity [8]. In addition to elevated risk of mortality, patients with frailty are also more likely to have peri-operative complications, such as myocardial infarction, pulmonary embolism, stroke, and surgical site complications [9].

Amongst non-surgical hospital admissions, moderate and severe frailty were also found to be associated with a higher rate of 30-day hospital re-admission compared to non-frailty (24.1% vs. 46%) [10]. Community dwelling adults with frailty are also significantly more likely to have reduced quality of life [11] and to need assistance with activities of daily living [12].

Cardiovascular disease remains the leading cause of death in the United States and has been projected to affect greater than 61% of the population by 2050 [13]. In a large prospective cohort study of 340,541 participants, Shi et al. demonstrated that both prefrailty and frailty were independently associated with an increased risk of incident heart failure, with hazard ratios of 1.40 and 2.07, respectively, compared to non-frail individuals [14]. Another prospective cohort study of Medicare beneficiaries aged greater than 65 years from the National health and Aging Trends Study without history of coronary heart disease showed that at 6 year follow-up, frailty was associated with higher risks of myocardial infarction [hazard ratio (HR) = 1.95], adverse cardiovascular events (HR = 1.59), stroke (HR = 1.71), and death (HR = 2.70) [15]. Given the substantial incidence of new-onset cardiovascular disease among frail individuals, these findings underscore the imperative to incorporate frailty assessment into cardiovascular disease management strategies, particularly in the realm of primary prevention.

Frailty plays a critical role in shaping multiple dimensions of care warranting thoughtful engagement and potential intervention by anesthesiologists. Frailty has been associated with a 1.5-fold increase in healthcare expenditures within the first year following major elective noncardiac surgery [16]. Given its strong association with increased peri-operative mortality, it is essential to incorporate comprehensive goals-of-care discussions that both address peri-operative and long-term care goals [17].

## 3. Pathophysiology of Frailty

**Figure 1:**
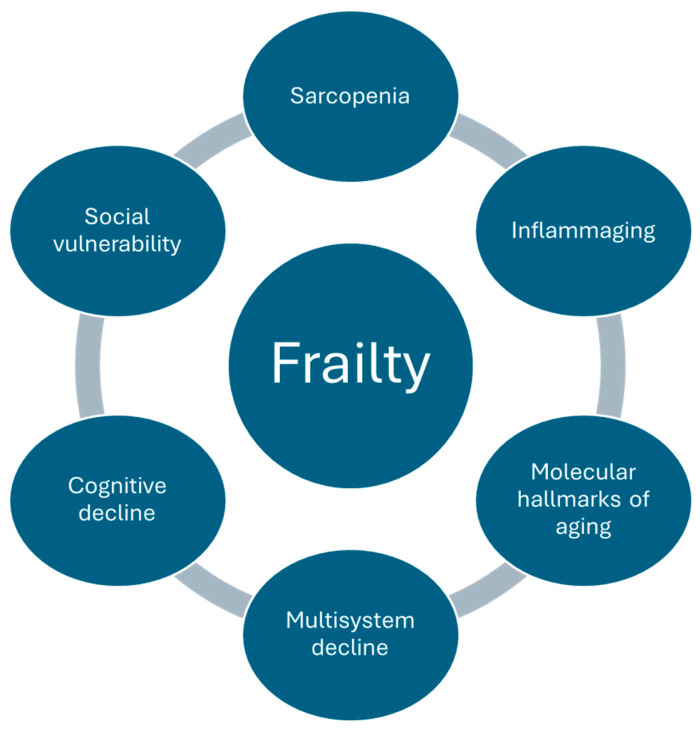
Summary of frailty pathophysiology.

Frailty is largely multifactorial in its cause and multidimensional in its impact (Figure 1) [18,19]. This is further supported by the fact that the frailty phenotype and frailty index often disagree on frailty classification for a substantial number of patients, despite both having clinical applicability and predictive value [20]. Its pathology is influenced by various processes such as inflammation, dysregulation of organ systems, sarcopenia, oxidative stress, and cellular and molecular signaling pathways.

The concept of “inflammageing” describes the long-term systemic inflammation that arises with aging [21,22]. Among the many examples of how inflammageing correlates with poor health outcomes, cardiovascular disease (CVD) remains one of the more consequential. The discussion on whether inflammatory cytokines causally create cardiovascular pathologies or those markers are instead an indicator of an underlying pathology is still underway. Nevertheless, those two conclusions may not be mutually exclusive, and thus some degree of self-amplification may exist regarding the relationship between inflammation and mortality secondary to CVD [22]. Likewise, it is argued that a similar cyclical mechanism may underlie the other pathologies that are seen with inflammaging, such as kidney disease, diabetes mellitus, cancer, sarcopenia, and many others [22]. Furthermore, the question still remains of whether attempts at treatment of the inflammatory processes that occur with aging can help prevent the onset of frailty. The accumulation of damage-associated molecular patterns (DAMPS) from injured cells within organ systems such as the gut, cardiovascular system, and virtually all others, include reactive oxygen species from damaged mitochondria which add to the process of inflammaging [22,23].

Cellular senescence describes a state of cell cycle arrest and permanent cessation of cellular division. While it serves a biological function in cancer suppression and tissue repair, it is thought to play an important role in aging and frailty [24]. Cellular senescence has been identified in association with advanced age in skin, pancreas, kidney, brain cortex, liver, spleen, and intestine [25]. Senescence cells are thought to have a distinct secretory profile that include chemokines, interleukins, and growth factors which induce deleterious effects on the local microenvironment [25]. This “secretome”, termed senescence-associated secretory phenotype (SASP), has been associated with physical frailty and sarcopenia in older patients [26,27].

Another phenomenon typically associated with frailty is the loss of functional stem cell reserve. Bombelli et al. reported that compared to non-frail older adults, frail adults had significantly higher proportion of renal and hematopoietic stem cells which demonstrated DNA damage and impaired function. This was associated with activation of oxidative stress and mitochondrial dysfunction [28]. The authors hypothesized that inflammation and oxidative stress could induce stem cell exhaustion [28].

Dysregulation of organ systems and microscopic processes has also been investigated in its relationship to the genesis of frailty. Loss of responsiveness and functionality of the autonomic nervous system has been seen with examples of reduced heart rate variability, orthostatic compromise, and general cardiac function [21,29,30]. Once again, accumulation of inflammatory biomarkers are correlated with increases in these losses of function [30].

Sarcopenia, generally defined as the reduction in both muscle mass and function, is a central aspect to frailty progression [31]. Like many of the concepts associated with frailty, it is also likely multifactorial in its genesis and may vary from patient to patient, often resulting in loss of independence [31]. It is believed to be largely the result of anabolic resistance, hormonal decline, catabolic processes (some of which may be inflammatory in nature), mitochondrial dysfunction, and inactivity [21]. C reactive protein (CRP), interleukin-6 (IL-6), and tumor necrosis factor- α (TNF-α) are implicated in its pathogenesis as well [21].

There are several approaches for measuring lean body mass and the presence of sarcopenia. Bioelectrical impedance analysis (BIA) is a simple point of care technique based on the electrical resistance differences between tissue types [32]. It is cheap, easy to perform, and has no radiation exposure. However, BIA performance is affected by patient’s hydration status or recent activity and measurement imprecision [33,34]. Radiological techniques such as magnetic resonance imaging (MRI), computed tomography (CT), and dual X ray absorptiometry (DXA) may also be used, with DXA typically considered a diagnostic gold standard [34,35]. Notably DEXA and BIA have reported comparable performance in sarcopenia identification, depending on the cutoff [34,36,37]. Thus, BIA could be considered an alternative for screening if DXA is not available.

Given the multidimensional nature of frailty, it is difficult to determine the exact causal relationship behind the different processes. For example, chronic inflammation may lead to sarcopenia and higher comorbidity burden (such as CVD risk) but itself may be a manifestation of other underlying biological processes. Eventually, as these individual systems and their redundancies deteriorate, a threshold is crossed as physiologic homeostasis is disrupted, leading to a “cacophony of multisystem dysregulation”, which synergistically results in the progression of frailty [19].

Beyond the biochemical and physiological changes that characterize frailty, it is also important to recognize the role of social vulnerability in frailty. A recent systematic review found that social vulnerability (loneliness, low social support, social isolation) was consistently associated with worsening physical frailty, while the presence of both synergistically worsened outcomes including survival [38].

## 4. Clinical Measures of Frailty

Various clinical measures have been developed to identify patients with frailty. The frailty index is one of the most commonly used tools to measure frailty, and it has been shown to correlate with increased vulnerability in older populations including risk of worsening health status, institutionalization, and death [18]. In this model, the presence and severity of frailty are determined by accumulated age-related health deficits [18,39]. Individual frailty index models vary but, in general, they include the consideration of 30 or more deficits. Included variables are deficits associated with health status that increase in prevalence with age. The deficits as a group cover a wide range of systems, such as activities of daily living (ADLs), instrumental activities of daily living (IADLs), mood, cognition, and presence of various chronic health conditions [1]. The frailty index is reported as a ratio of the patient’s deficit burden compared to the total number of factors considered [1]. Frailty indices can score variables in a dichotomous or ordinal fashion, though recent studies have shown that ordinal variables have a slight advantage in predicting all-cause mortality [40]. The disadvantage of this method is that it is time-consuming and requires a large amount of data. Therefore, it is not routinely used in clinical practice.

The frailty phenotype (FP) takes a different approach. The FP defines frailty as a clinical syndrome using the following criteria: unintentional weight loss, self-reported exhaustion, weakness, slow walking speed, and low physical activity [40,41,42,43]. A score zero is considered robust; one to two is considered pre-frail; three or greater is indicative of frailty. The frailty phenotype performs similarly to the frailty index in predicting all-cause mortality and poor quality of life, though the FI is better at predicting functional impairment than the FP [40,42]. The FP is currently the most clinically relevant scale given its simplicity, standardized scoring, validity, and reliability. Many other frailty scales incorporate similar elements that are used in the FP.

The Clinical Frailty Scale employs an ordinal scale and relies on clinical judgment to determine overall frailty with simple descriptors [44]. It was originally developed as a seven-point scale, though this has been updated to a 9-point scale as of 2020 [45,46]. Patients are graded on a scale of 1 to 9, with one being labeled as “very fit” to nine being labeled as “terminally ill.” Each tier has a simple descriptor, and clinicians score patients by clinical judgment. While the lack of specific descriptive criteria introduces an element of ambiguity, its value lies in the ease of use [43]. It requires no additional testing, equipment, or time to screen patients for frailty with this tool. For these reasons, the Clinical Frailty Scale is very useful for the inpatient setting. When compared to the frailty index, the Clinical Frailty Score similarly predicts death or entry into an institution [45]. Additionally, it has high interrater reliability [45].

Measures of defined physical tasks have been used as a surrogate measure of frailty. The TUG involves rising from a chair of standardized height, walking a fixed distance of 3 m, turning, returning to the chair, and sitting down again [47]. In one recent study, the lowest quintile for the TUG was >11.8 s in men and >12.5 s in women [48]. It is simple and requires no specialized equipment. Therefore, the TUG can be easily performed in most clinical settings, especially outpatient practices. Additionally, it has been shown to correlate with both the FI and the FP when distinguishing frail from non-frail patients [47,48]. However, it is less useful for distinguishing patients with prefrailty from frailty [47].

A number of additional clinical frailty scales have been developed in recent years as academic interest in frailty has increased [49]. The Study of Osteoporotic Fracture (SOF) Index identifies frailty in women at a comparable rate to the frailty phenotype, and predicts risk of falls, disability, fracture, and death [50]. The FRAIL scale uses a 5-item questionnaire to screen for frailty—fatigue, resistance, ambulation, illness, and weight loss. This frailty measure has been validated in the middle-aged African American population [51]. The Vulnerable Elders Survey specifically identifies community-dwelling older adults that are at risk for functional decline and death [52]. The Edmonton Frail Scale assesses various domains—cognition, balance and mobility, mood, functional independence, medication use, social support, nutrition, health attitudes, continence, burden of medical illness, and quality of life—and has been validated to be reliable and feasible for use by non-geriatricians [53].

## 5. Circulating Frailty Biomarkers

### 5.1. Clinical Biomarkers

Given that frailty is a complex, multifactorial syndrome arising from the cumulative impact of multiple disease processes, it is difficult to identify a singular biomarker to aid in identifying frailty in clinical practice. Although there have been several biochemical and genetic markers identified that have been associated with frailty, it would be valuable to understand the utility of frequently obtained markers of organ function, such as creatinine, eGFR, cystatin C, BNP, albumin, and hemoglobin in our understanding of frailty (Table 1).

The glomerular filtration rate (GFR) is the primary diagnostic criteria in the diagnosis of chronic kidney disease (CKD) along with albuminuria and is a frequently obtained biomarker in clinical practice, typically estimated from serum creatinine [54]. In univariate analysis, creatinine and eGFR have been shown to have a positive and negative correlation with frailty, respectively; this correlation disappears in multivariable adjusted analyses [55]. Cystatin C is another marker of renal function that has growing clinical utility in its ability to minimize race-based calculations in typical estimated GFR calculations as well as the contribution of muscle mass to creatinine levels [56]. In a cohort study by Li et al., cystatin C and eGFRcys were associated with long-term frailty trajectories and physical function decline even when controlling for creatinine level [56]. Also relevant is the eGFRdiff which is the difference between the eGFR cystatin and eGFR creatinine, calculated as eGFRcys—eGFRcr. In a subset cohort analysis of 9092 hypertensive patients in the Systolic Blood Pressure Intervention Trial (SPRINT), Potok et al. concluded that each one-standard deviation higher in eGFRdiff was associated with 24% lower odds in frailty prevalence [odds ratio (OR) = 0.76] [57]. In addition to frailty, a greater eGFRdiff was associated with a lower incidence of injurious falls (HR = 0.84), cardiovascular events (HR = 0.89), and all-cause mortality (HR = 0.71) [57]. Another cohort analysis by Potok et al., found that those with a negative eGFR diff (greater eGFRcr than eGFRcys) had a nearly two-fold higher risk of poor functional status, as well as a 14-cm^2^ smaller thigh muscle area as seen on CT scan and found that it minimally attenuated the association [58]. Although Cystatin C is less affected by muscle mass than creatinine, some studies have shown that eGFR correlation with frailty can be attenuated when accounting for muscle mass. A cross-sectional analysis by Yuan et al. demonstrated that although eGFRdiff was positively associated with lower odds of frailty [OR = 0.63, 95% confidence interval (CI) = 0.45; 0.89], this association was rendered insignificant when adjusting for D_3_Cr muscle mass (OR = 0.85, 95% CI= 0.58–1.24) [59]. Although cystatin C is growing in popularity as a more accurate clinical measure of renal function, more research is needed on its utility to screen for frailty in the setting of low muscle mass or sarcopenia given conflicting results in prior studies.

Creatinine and cystatin C are just two of many commonly obtained clinical measures that have been implicated in frailty. B-type natriuretic peptide (BNP), frequently assessed in patients with heart failure, is a neurohormonal marker released by cardiac myocytes in response to ventricular wall stretch, fluid overload, and myocardial hypertrophy [60]. In a cross-sectional analysis of a prospective cohort study conducted by Yao et al., elevated BNP levels (≥100 pg/mL) were independently associated with increased odds of both prefrailty (OR = 1.61, 95% CI = 1.13–2.29) and frailty (OR = 2.63, 95% CI = 1.61–4.32) [60]. A multivariable analysis of the NSQIP database in patients undergoing spine surgery revealed a significantly higher prevalence of hypoalbuminemia among frail individuals (11.4%) compared to their non-frail counterparts (4.3%) [61]. Frail patients with hypoalbuminemia exhibited markedly increased odds of surgical complications (OR = 5), reoperation (OR = 3.3), hospital re-admission (OR = 3.1), and mortality (OR = 31.8) relative to those without hypoalbuminemia [61]. A cross-sectional study examining adults older than 65 in Singapore found that for each 1 g/dL increase in hemoglobin there was a 6% decrease in frailty odds after covariable adjustment, but the absolute prevalence of anemia in the frail group was 28.6% compared to 12.8% in the robust group [61].

Although several routinely measured clinical biomarkers such as BNP, albumin, and hemoglobin have been associated with frailty, the overall prevalence of clinically detectable abnormalities among frail individuals remains modest. This underscores the limitation of relying on any single biomarker for the identification of frailty and highlights the need for a more comprehensive or multimodal approach to effectively detect and stratify at-risk individuals.

### 5.2. Metabolic Profile and Metabolomics

Recent evidence suggests that metabolic dysfunction plays a significant role in the development of frailty. Metabolic syndrome, a cluster of conditions, including hypertension, dyslipidemia, hyperglycemia, and central obesity, has been closely linked with increased frailty risk. A longitudinal study by Zeng et al. found that greater metabolic syndrome (MetS) severity was associated with a 20.5% increased risk of frailty. Additionally, each 1-standard deviation (SD) increase in MetS score was associated with faster progression of the frailty index (FI), suggesting that metabolic health may directly influence frailty trajectory [62].

Metabolomics, the comprehensive analysis of metabolites within biological systems, offers a new approach to study the biochemical signatures associated with frailty by determining phenotypic signatures of metabolic derangements that are associated with disease states. Multiple metabolic processes have been implicated in frailty. A recent study implicated dysregulation of lipid metabolism, vitamin E metabolism, and the carnitine shuttle in frail patients. The investigators used liquid and gas chromatography–mass spectrometry metabolomics in the study of over 1000 participants. Two chemical classes, tocotrienols and carnitines, exhibit significantly modulated under-expression when overlaid across the frailty index [63].

Carbohydrate metabolism has also been shown to be affected by frailty. Westbrook et al. compared the serum metabolomes of younger compared to older populations and older robust compared to older frail populations. In frail subjects (as defined by a frailty phenotype screening tool), glucose and pyruvate were elevated while Glucose-6-Phosphate and Glycerol-3-Phosphate were lower compared to non-frail and young subjects [64].

The tricarboxylic acid metabolism also experienced significant changes in its metabolome in frail subjects compared to younger and non-frail subjects. In the same study by Westbrook and colleagues, serum *cis*-aconitate, oxaloacetate, and beta l-citryl-glutamate were elevated in frail subjects compared to non-frail and young subjects [64]. Neurotransmitter production and metabolism are also affected by frailty. Serum levels of glutamate, gamma-aminobutyric acid (GABA), and aspartate (a precursor for *N*-acetyl-aspartyl-glutamate) were significantly elevated in frail subjects compared to both young and non-frail subjects. Interestingly, some of these findings have also correlated with other circulating biomarkers of frailty. For example, α- ketoglutarate, N-acetyl-aspartyl-glutamate (NAAG), and pyruvate were positively correlated with IL-6. Additionally, NAAG, glutamate, GABA, oxaloacetate, glucose, *cis*-aconitate, aspartate, malate, and beta l-citryl-glutamate were positively correlated with TNFαR1 levels [64]. These findings support the hypothesis that metabolic dysregulation plays a role in the pathogenesis of frailty.

Differences in amino acid metabolism have also been seen in frail versus non-frail patients. Calvani et al. studied circulating amino acid levels in robust controls, adults with frailty and sarcopenia, and frail adults with type 2 diabetes mellitus. In adults with frailty and diabetes, the amino acid profile was characterized by higher levels of 3-methylhistidine, alanine, arginine, ethanolamine, and glutamic acid. In adults with frailty and sarcopenia, the amino acid profile showed higher levels of aminoadipic acid, asparagine, aspartic acid, cystine, taurine, and tryptophan [65].

As with inflammatory markers, individual metabolic products are likely to be highly variable between individuals and of low diagnostic values. However, omics technologies enable the mapping of metabolic profiles associated with frailty. This allows for potential biochemical diagnosis of frailty but also facilitates better understanding of the biochemical processes which underline frailty. The reason for patterns of metabolic disturbances seen in frail patients is likely to be multifactorial. Nutritional status is one possible explanation given the involvement of catabolic pathways, and poor nutrition status is strongly associated with frailty [66]. Another possible cause of metabolomic changes in frailty is mitochondrial dysfunction [66]. Disruptions in the balance of individual metabolites could lead to a cascade of pathological processes leading to the development of frailty.

### 5.3. Genetic and Epigenetic Markers

The pathophysiology of frailty is complex involving multiple endocrine, inflammation, and metabolic pathways which leads to great potential in incorporating genetic markers implicated in these processes to aid in identifying at-risk individuals and guide interventions. There are many components of genetic expression that potentially contribute to frailty phenotypes including individual DNA polymorphisms, RNA expression, and DNA modulation such as histone methylation. A large genome-wide association study (GWAS) by Ye et al., a total of 8,883,488 single nucleotide polymorphisms (SNPs) were examined, and subsequently 37 independent novel risk loci for frailty were identified, including seven not previously described [67]. Further analysis using Multi-biomarker Analysis of GenoMic Annotation (MAGMA) and Linkage Disequilibrium Score Regression (LDSC) revealed a significant association between these genetic markers and frailty in brain tissue [67]. Of the novel loci identified, many are located near protein-coding genes, including actin regulating proteins, protein kinase signal transduction, and METTL16, a methyltransferase involved in RNA modification [67].

Exosomes are small cell-derived vesicles found within extracellular fluids and originate when cellular multivesicular bodies fuse with plasma membranes [68]. MicroRNAs present in these exosomes have been of interest as a potential marker of frailty given their ability to alter cellular activity and behavior. MicroRNAs are short, non-coding RNA molecules that modulate gene expression by acting at the post-transcriptional level. One study isolated plasma from young, robust older, and frail (as defined by frailty phenotype) older individuals. Authors identified eight unique plasma miRNAs elevated in frail adults (miR-10a-3p, miR-92a-3p, miR-185-3p, miR-194-5p, miR-326, miR-532-5p, miR-576-5p, and miR-760) [69]. Another study examined exosomes present in saliva and ultimately found that miR-24-3p was associated with aging. It was also demonstrated that saliva has a different miRNA expression as compared to serum [70].

MiRNA has also been associated with change in peri-operative outcomes. A case–control study found that patients who developed postoperative delirium had higher expression of miR-320 [71]. In another study, a panel of 3 microRNAs (miR-122-5p, miR-192-5p, miR-151a-5p) have superior predictive accuracy for posthepatectomy liver failure compared to traditional liver function tests [72].

Similarly, mitochondrial microRNAs (mitomiRs), such as miR-21, miR-126a-3p, and miR-146a-5p, have been implicated in aging and age-related diseases such as frailty by promoting mitochondrial dysfunction through Bcl-2 downregulation, leading to impaired autophagy, increased oxidative stress, inflammation, and apoptosis [73]. In particular, plasma c-miR-21 has been shown to be increased in frail adults compared to similarly aged, robust adults without frailty [74]. MiR-21 has been implicated in multiple inflammatory pathways involving TGF-β, where high miR-21 is associated with increased inflammation [75]. Healthy centenarians were observed to have comparable miR-21 levels as healthy 20-year-old subjects [75].

There is a growing body of evidence that suggests that epigenetic regulation plays a critical role in the development and progression of frailty, which can aid in bridging the gap between genetic characteristics, environmental exposures, and clinical phenotypes. DNA methylation is an epigenetic modification in which there is an addition of a methyl group to a cytosine base, typically followed by a guanine base, called a CpG site. In a meta-analysis by Mak et al. examining epigenome-wide association studies from the Swedish Adoption/Twin Study of Aging (SATSA) and the Longitudinal Study of Aging Danish Twins (LSADT), of 829 participants, 589 CpG sites were identified as being associated with frailty [76]. Of these, cg05582310 and cg27304020 were found to have the greatest statistically significant associations with frailty, in which both are hypomethylated with elevated frailty scores [76]. Alternatively, they also found two genes that when hypermethylated were associated with an increased frailty index, MIR596 (microRNA-596), and TAPBP (tapasin) genes [76]. Tapasin is a glycoprotein that is involved in immune system antigen presentation, and hypermethylation has been associated with obesity and insulin sensitivity, while MIR596 hypermethylation has been associated with tumor development, suggesting pathways related to immune modulation and cancer may be associated with frailty [76]. A GWAS meta-analysis by Atkins et al. examining the frailty index in UK Biobank participants and Swedish TwinGene participants found a lower than previously estimated SNP-based heritability at 11% [77]. Of the 14 loci identified, 2 had the most robust significant rs9275160 (associated with HLA-DQB1 and HLA-DQA2) and rs82334 (nearest gene HTT) [77]. HLAs (Human Leukocyte Antigens) are cell-surface proteins that regulate immune function, which are well-known to decline with age. DNA methylation of the TNXB/TNXA/STK19 (Tenascin XB/puta-tive Tenascin XA/Serine/threonine-protein kinase 19) gene cluster in the HLA region has been linked to higher frailty [77]. Further analysis showed an association between frailty and a downregulation of several genes linked to neuronal function, including ANK3 (Ankyrin), a scaffold protein involved in neurotransmission, and NLGN1 (Neuroligin 1), a synapse cell-surface protein.

While our understanding of the genetic contribution to frailty is currently advancing, it remains a promising body of biomarkers that warrants further research to identify its clinical utility.

### 5.4. Inflammatory Markers

The underlying pathophysiology of frailty is not completely understood, though various mechanisms are thought to be related to its development. Inflammageing, and the associated age-related increase in proinflammatory cytokines, is thought to be one of the major contributors to the pathogenesis of frailty [78]. Various inflammatory markers have been studied in relation to frailty. A systematic review and meta-analysis by Xu et al. evaluated 53 cross-sectional studies relating to the relationship between inflammatory biomarkers and frailty [79]. The biomarkers that were studied include CRP, leukocyte levels, lymphocyte levels, IL-6, IL-10, and TNF-α. The results showed that CRP and TNF-α levels were significantly increased in the frail group compared to the pre-frail and robust groups. Of note, CRP is nonspecific and can be elevated in many pathophysiologic conditions. IL-6 levels were significantly increased in both the frail and pre-frail groups compared to the robust group. Lymphocyte level, but not overall leucocyte level, significantly decreased in the frail and pre-frail groups compared to the robust group. IL-10 levels were not significantly changed between groups [79]. One study also found an association between frailty and increased IL-4 and interferon- γ (IFN- γ) in addition to high-sensitivity CRP and TNF-α [80]. There is some evidence for a genetic basis for frailty. Mekli et al. showed that single nucleotide polymorphisms (SNPs) in the promoter region of the TNF-α gene on chromosome 6 were associated with increased frailty [81].

Many studies on frailty exclude participants with neurocognitive disease and those living in long-term care facilities to avoid confounding results. However, these groups represent a significant part of the frail population. Collerton et al. specifically studied biomarkers of frailty in adults aged 85 years and older and included those in long-term care as well as those with neurocognitive deficits [82]. This study confirmed that high CRP level was associated with greater frailty risk. Interestingly, elevated neutrophils were also associated with increased frailty risk. Lower basal levels of IL-6 and TNF-α were associated with lower frailty risk. This study also found that decreased albumin level and total lymphocyte level were associated with increased risk of frailty [82]. Langmann et al. studied biomarkers of frailty in 178 women ≥ age 65 with osteoporosis in living in long-term care facilities. In this study, frail participants had significantly higher levels of HS-CRP, TNFα-R1, TNFα-R2, IL-6, and IL-6-sR than non-frail participants [83].

Longitudinal studies have shown some differences in the significance of each biomarker compared to cross-sectional studies. The Longitudinal Aging Study Amsterdam also confirmed an association between elevated CRP level and increased risk of frailty over the 3 year study period; however, IL-6 did not share this association [84]. The China Health and Retirement Longitudinal Study also showed that increased baseline high-sensitivity CRP was associated with a 1.18 times increased risk of developing frailty over the 3-year follow-up period [85]. Walker et al. conducted a longitudinal study spanning 24 years. In this study, midlife increased CRP was associated with increased risk of developing frailty later in life. Each one-standard deviation increase in initial inflammation composite score was associated with 32% higher odds of frailty and a greater number of frailty characteristics at 21 years of follow-up [86]. This suggests that systemic inflammation may be involved in the pathogenesis of frailty decades prior to its clinical onset. While individual inflammatory markers are likely to be of low specificity for frailty, patterns of inflammatory marker change could be a potential biochemical marker of frailty.

Growth/differentiation factor 15 (GDF-15) is a pleiotropic cytokine from the Transforming Growth Factor β (TGF β). It is expressed by various tissue types; cellular stressors are thought to increase GDF-15 expression [87]. While transient GDF-15 elevation has been observed in relation to physical exercise, elevated resting GDF-15 has been observed in association with lower muscle strength [88]. In a secondary analysis of over 1000 patients, elevated GDF-15 was associated with both the presence of frailty as well as sarcopenia [89]. Another large study found that after adjusting for confounders such as age, comorbidity, and malnutrition, GDF-15 remains independently associated with sarcopenia [90].

### 5.5. Markers of Oxidative Stress

Oxidative stress also plays a significant role in the cellular and organ system changes associated with frailty [91]. Dysregulated generation of reactive oxygen species could be detected through the identification of oxidation products such as isoprostanes, malondialdehyde (MDA), and 8-hydroxy-2′-deoxyguanosine, as well as changes in enzyme expression such as lipoprotein phospholipase A2 (Lp-PLA2).

In a cross-sectional study of over 1900 participants, frailty phenotype was associated with higher isoprostanes and Lp-PLA2, as well as slower gait speed [92]. Similarly, another study of over 700 patients reported that frailty phenotype was associated with higher MDA level and protein oxidation [93].

Vitamin E, a fat-soluble antioxidant, has also been implicated in frailty. Reduced dietary intake of vitamin E [94] and reduced plasma vitamin E level [95] have both been associated with higher incidence of frailty phenotype. Other antioxidants that may play a critical role in preventing or delaying the onset of frailty are glutathione, cysteine, and homocysteine. The plasma total thiol test measures an individual’s antioxidant capacity via these thiol compounds and has shown some correlational utility when studied in frail individuals [91,96].

### 5.6. Multi-Biomarker Approach and Machine Learning

Since frailty involves a complex network of interconnected biochemical processes and demonstrates considerable inter-individual variability, it is unlikely that single-marker approach to screening will yield meaningful diagnostic performance at a population level. Conversely, Sajeev et al. reported that a panel of demographic (gender), biochemical (hemoglobin, creatinine, CRP) and healthcare informatics parameters (emergency room triage classification) can be used to identify frail patients amongst an inpatient population [97].

Machine learning also has potential to not only help identify patients at risk of frailty, but also the specific biomarkers, metrics, or demographic factors most associated with frailty. By comparing traditional factors used to diagnose frailty to factors identified through machine learning, one study found that machine learning models had better concordance with frailty markers such as strength, nutrition, and mobility [97]. However, machine learning models were also able to identify more subtle factors such as anthropometrics, lifestyle, distress, and environment [97]. Other work using machine learning has discovered novel genetic and epigenetic risk factors and loci associated with increased risks of frailty, such as APOE and CDKN2B [98]. Furthermore, machine learning models also identified exploratory markers such as vitamin D, gamma glutamyl transferase (GGT), fibroblast growth factor 21 (FGF21), and neurofilament light chain (NfL) in association with frailty [98,99]. Lastly, machine learning approaches could identify frailty based on clinical parameters such as hemoglobin, neutrophil ratio, GFR, BUN, Cr, and potassium [98,100].

These results all suggest machine learning should be employed to discover previously underutilized aspects of a patient’s history to help identify frailty. These features can be much more accessible to clinicians in practice and are often already available from previous labs. As such, machine learning should also continue to be utilized to help uncover other factors that may not yet be used in identifying frailty.

**Table 1:** Summary of biochemical markers of frailty.

Category	Findings/Associations
**Clinical markers**	eGFRcys and eGFRdiff may be more predictive of frailty than eGFRcr [56] Low hemoglobin, low albumin, and elevated BNP are each found in a subset of frail patients [60,61]
**Metabolic profile (metabolomics)**	Dysregulation of **glycolysis**: ↑ glucose & ↑ pyruvate; ↓ glucose-6-phosphate and glycerol-3-phosphate [64] Alterations in the **TCA cycle**: ↑ cis-aconitate, α-ketoglutarate, oxaloacetate, β-l-citryl-glutamate [64] Perturbations in **lipid metabolism**: decreased tocotrienols and elevated acyl-carnitines [63] Altered **amino acid profiles** in frail/sarcopenic individuals: ↑ 3-methylhistidine, alanine, arginine, glutamic acid; ↑ aminoadipic acid, taurine, tryptophan [65]
**Genetic and Epigenetic Markers**	**Elevated plasma miRNAs** in frail older adults: miR-10a-3p, miR-92a-3p, miR-185-3p, miR-194-5p, miR-326, miR-532-5p, miR-576-5p, miR-760 [69] **Elevated** mitochondrial miRNAs associated with frailty: **miR-21**, miR-126a-3p, and miR-146a-5p [73,74] Identification of **SNPs** through genomic studies [67] **DNA methylation**: cg05582310, cg27304020, rs9275160 and rs82334 [76]
**Oxidative stress**	**↑ Isoprostanes, ↑ MDA, ↑ Lp-PLA_2_ activity** associated with frailty phenotype [93] **↓ Antioxidant capacity** (measured by total thiols) via glutathione, cysteine, homocysteine, correlates with frailty [96] **Low plasma vitamin E** is linked to higher frailty risk [94]
**Inflammatory markers**	**↑ CRP & ↑ IL-6** consistently linked with frailty and prefrailty [79,82] **↑ TNF-α** and its soluble receptors moderately associated with frailty in some cohorts [82] **↓ lymphocyte counts** and **↑ neutrophils** also reported as frailty-related changes [79] **↑ GDF-15** associated with frailty [89,90]

BNP: brain natriuretic peptide, CRP: C reactive protein, eGFR: estimated glomerular filtration rate, eGFRcys: cystatin-derived eGFR, eGFRcr: creatinine-derived eGFR, eGFRdiff: difference between eGFRcys and eGFRcr. IL: interleukin, Lp-PLA2: lipoprotein phospholipase A2, MDA: malondialdehyde, miRNA: micro-RNA, SNP: single nucleotide polymorphism, TCA: tricarboxylic acid (cycle), TNF-α: tumor necrosis factor α.

## 6. Radiological Markers of Frailty

With alterations in body composition being one of the hallmarks of frailty, various medical imaging modalities have been employed to detect these changes and use this data to both define and risk stratify frail patients. With the common clinical manifestation of frailty being an impairment in mobility and balance, much of the focus has been on skeletal muscle. Originally defined as a loss of muscle mass, sarcopenia associated with frailty now functions as an umbrella term encompassing muscle size, composition, and strength (dynapenia) [101]. Though not used frequently in the peri-operative setting, DXA is a well-accepted common imaging modality that can measure body composition including bone (and its density), adipose tissue, and lean body mass. Appendicular (limb) muscle mass is frequently corrected for height and DXA measurements are commonly used clinically for frailty assessments [102]. Unfortunately, the technology has limitations based on intra-machine variability, patient hydration status, and the inability to measure the distribution and composition of lean body mass [33].

CT scanning is a frequently used cross-sectional clinical imaging modality for diagnosis and planning of surgery. Opportunistic radiographic data on these scans can not only serve as a biomarker for frailty but can predict post-surgical outcomes. When CT scans are obtained for diagnostic purposes, they are focused on particular body areas of interest and may not include axial or appendicular areas that DXA has traditionally used for assessments of skeletal muscle. Extrapolations from muscle cross-sectional area on CT scan at the third lumbar level can be corrected for both height and BMI to provide a reliable estimate of relevant skeletal muscle mass [103].

While skeletal muscle cross-sectional area and mass have a correlation with frailty, muscle composition appears to be a strong clinical predictor of both clinical muscle strength as well as patient outcomes. Normal aging is associated with an increase in the adipose tissue associated with muscle, both within the muscle itself and within groups of muscles, a process known as myosteatosis. When imaged by CT scan, the more adipose tissue a muscle contains, the less dense it appears radiographically (lower Hounsfield Units). Myosteatosis has long been known to affect muscle strength, independent of muscle size [104], and when followed longitudinally, high intramuscular fat correlates with a patients decline in gait speed [105]. Muscle radiodensity, but not muscle size, correlated with TUG test and Grip Strength (as surrogate measures of frailty) [106,107]. Grip strength has even correlated with myosteatosis in distant muscle groups such as the thigh [108].

Radiographic muscle biomarkers are not only associated with frailty score, but also mortality in community-dwelling elderly as CT-derived parameters of total muscle mass (cross-sectional area and radiodensity) correlate with all-cause mortality at one year [109]. Long term observations show that myosteatosis is associated with an 8% increase in mortality with the primary causes of death being cardiovascular and cancer on eight-year follow up [110]. For Major Adverse Cardiac Events, long-term follow-up has shown that the presence of myosteatosis rivals the risk associated with diabetes [111]. Frail patients that experienced myocardial infarction had a 12% increase in mortality for each one-point increase in Clinical Frailty Score [112]. The trajectory of chronic disease states such as cirrhosis, hospital admissions, liver decompensation, and mortality are all correlated with myosteatosis/sarcopenia [113,114]. For patients living with Human Immuno-Deficiency Virus, myosteatosis is correlated with coronary artery plaques [115].

Obesity is one of the most prevalent chronic medical conditions and its impact on the elderly and frail elderly has unexpected findings. A well described “obesity paradox” exists where obese elderly patients appear to have a survival advantage when faced with other chronic medical conditions such as heart failure, diabetes, and pulmonary disease [116,117,118]. However, obesity is not a uniform disease process, and the combination of obesity and sarcopenia has a prevalence as high as 12% in older people in North America [119] and is associated with decreased survival for disease states such as cancer [120] yet appears to have no outcome effect on cardiovascular disease [121] or kidney transplantation outcomes [122].

In the acute disease setting, the presence of frailty markers has been shown to correlate negatively with outcomes. In elderly patients undergoing hospital admission for active COVID-19 infection, Myosteatosis combined with inflammatory markers such as IL-6 is highly predictive of mortality [123]. In patients with acute stroke, myosteatosis of the masseter muscle correlates with mortality after successful reperfusion [124]. In elderly patients undergoing acute abdominal surgery, myosteatosis and grip strength were correlated with non-home discharge and days alive outside of hospital [125].

When used in the peri-operative setting, CT-derived frailty biomarkers have consistently been shown to correlate with an increased complication rate. In one study of 48,444 patients undergoing abdominal surgery, CT-derived muscle assessments of frailty correlate with mortality, 30-day re-admission and NSQIP measured complications [126]. For elderly patients undergoing Trans Catheter Aortic Valve Replacement (TAVR), muscle cross-sectional area and density have been shown to correlate with both short and long-term mortality [127,128] and can be better predictors than clinically derived frailty scores [129]. In vascular surgery patients undergoing peripheral bypass, CT-derived biomarkers of sarcopenia are equally as predictive of one year mortality as a Clinical Frailty Score [130]. A similar pattern of increased myosteatosis and decreased muscle mass on pre-operative imaging is predictive of decreased survival for endovascular aortic procedures as well [131,132]. For patients undergoing renal transplantation, myosteatosis is associated with increased mortality, but low muscle mass as measured by CT cross-sectional area is not [133].

For cancer patients undergoing resection, both muscle mass and radiodensity correlate with length of stay, anastomotic leak, and short- and long-term mortality in colon cancer surgery [134,135,136]. In both gastric and esophageal cancer resection, myosteatosis correlates with anastomotic leaks, short-term complications, and long-term 5-year survival [137]. For patients with head and neck cancers undergoing resection, sarcopenia identified pre-operatively was associated with overall survival time approximately one-quarter of that of patients without sarcopenia on pre-operative evaluation [138].

Other imaging modalities such as ultrasound and MRI may be less common in the peri-operative period but are able to detect body composition changes associated with frailty. Ultrasound measurements of quadriceps muscle depth correlated with traditional CT-derived muscle biomarkers and were predictive of post-operative discharge to a skilled nursing facility [139]. However, with the echo intensity of skeletal muscle approximating that of adipose tissue, assessments of myosteatosis may be difficult using this modality. MRI can evaluate both muscle area and myosteatosis with similar parameters as validated by CT imaging. However, MRI offers an advantage of evaluating specific brain changes associated with frailty. When looking at community-dwelling adults, the severity of frailty correlated with total white matter hyperintensity and low gray matter volume [140]. Other components of physical frailty such as weakness are correlated with loss of gray matter in the hippocampus, amygdala, and fusiform gyrus, while slowness was additionally correlated with medial prefrontal and orbitofrontal cortex [141]. However, as brain MRI may not be a common pre-operative imaging modality, its use as a biomarker in the peri-operative setting is limited.

## 7. The Future of Early Frailty Intervention

Effective screening and early recognition of frailty can facilitate meaningful interventions that improve patient outcomes. In an early scoping review of community-dwelling older adults, Apóstolo et al. reported that multimodal interventions including physical exercise, nutrition, and memory training could slow the progression of frailty while being cost-effective [142]. A more recent clinical trial of older community-dwelling adults also demonstrated that multimodal interventions reduced the incidence of frailty [143]. Physical exercise typically consisted of a regimen of resistance exercises for various muscle groups, flexibility, balance, and mobility exercises [143]. Dietary interventions included daily protein and other dietary supplementation, as well as nutritional goal setting [143].

Several small sample sized studies have applied multimodal interventions to frail patients such as exercise, nutrition, and psychological support. Not surprisingly, exercise interventions for total knee arthroplasty were able to improve various exercise measured post-operatively such as strength and TUG [144].

For frail patients with cancer, several studies have shown the benefits of prehabilitation. For lung cancer patients, intense prehabilitation for seven days has been shown to improve pre-operative physical functions, which correlated with reduced pulmonary complications and length of stay post-operatively [145]. Other prehabilitation intervention studies reported mixed results of improved functional capacity or post-operative outcomes [146,147].

**Figure 2:**
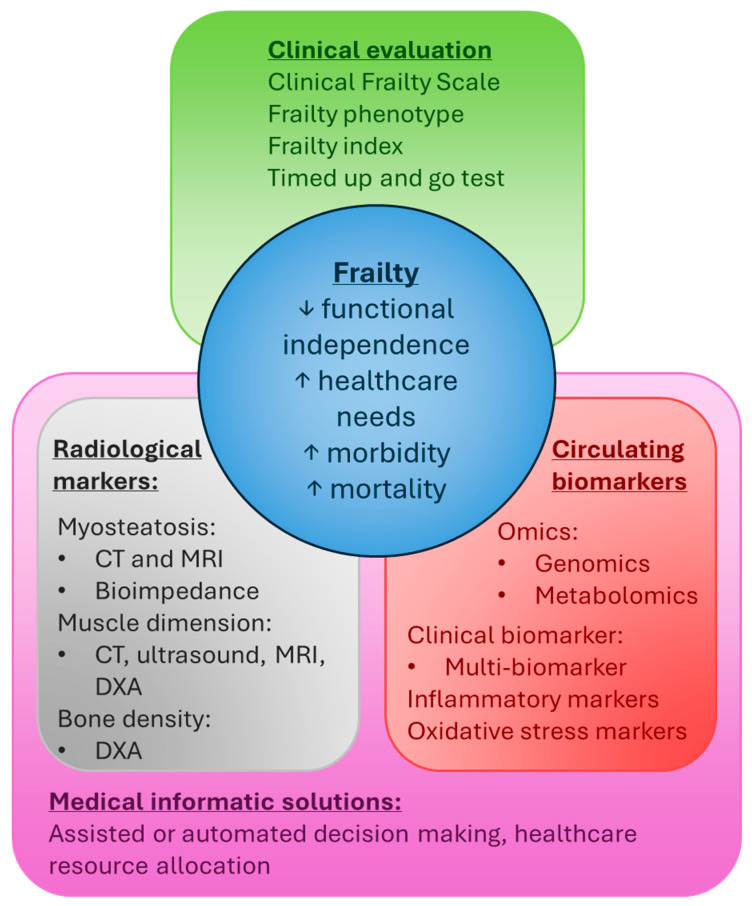
Summary of frailty recognition and outcomes.

Given the potential benefits of early frailty intervention, there is a significant clinical need for effective population-level frailty screening. A multifaceted approach that leverages biochemical markers, opportunistic imaging, and digital health data could complement the infrastructure for clinical frailty evaluation, providing better accessibility and create more opportunity for early intervention (Figure 2). To implement this real-world healthcare, there needs to be an informatics infrastructure that can bring together information from electronic medical records, medical imaging systems, and clinical decision-making tools.

## 8. Conclusions

In summary, frailty is a complex biological, psychological, and social process. While clinical screening and diagnosis are the current gold standard, it is not without its limitations. Opportunistic or deliberate radiological evaluation could provide valuable insight that can facilitate the recognition of frailty. Better understanding of the biochemical processes associated with frailty could lead to the development of biochemical assays that offer alternative options for screening.

Finally, there is an urgent need for larger clinical studies that identifies and validates frailty biomarkers, as well as feasibility studies of an integrated approach to frailty screening. By combining biomarkers, imaging, and informatics systems in a smart, connected way, frailty could be detected and intervened early, which have demonstrated efficacy in improving patient outcomes.

## Data Availability

No new data was created for this study. Studies cited in this review may be found in the references section.

## Abbreviations

The following abbreviations are used in this manuscript:

ADLs	Activities of daily living
CRP	C reactive protein
CVD	Cardiovascular diseases
CT	Computed Tomography
DAMPS	Damage-associated molecular patterns
DXA	Dual X ray Absorptiometry
FP	Frailty Phenotype
GAMA	Gamma-aminobutyric acid
GDF-15	Growth/differentiation factor 15
HGS	Hand grip strength
IFN- γ	Interferon- γ
IL	Interleukin
Lp-PLA2	Lipoprotein phospholipase A2
MetS	Metabolic Syndrome
MRI	Magnetic Resonance Imaging
MDA	Malondialdehyde
miRs	microRNAs
mitomiRs	mitochondrial microRNAs
NAAG	*N*-acetyl-aspartyl-glutamate
NSQIP	National Surgical Quality Improvement Program
SNP	Single nucleotide polymorphisms
TGF	Transforming Growth Factor β
TUG	Timed up and go test
TNF-α	Tumor necrosis factor-α
VASQIP	Veterans Affairs Surgical Quality Improvement Program

## References

[1] Searle S.D., Mitnitski A., Gahbauer E.A., Gill T.M., Rockwood K. (2008). A standard procedure for creating a frailty index. BMC Geriatr..

[2] Walsh B., Fogg C., Harris S., Roderick P., de Lusignan S., England T., Clegg A., Brailsford S., Fraser S.D.S. (2023). Frailty transitions and prevalence in an ageing population: Longitudinal analysis of primary care data from an open cohort of adults aged 50 and over in England, 2006–2017. Age Ageing.

[3] The Older Population: 2020. https://www2.census.gov/library/publications/decennial/2020/census-briefs/c2020br-07.pdf.

[4] Hall M.J., DeFrances C.J., Williams S.N., Golosinskiy A., Schwartzman A. (2010). National Hospital Discharge Survey: 2007 Summary.

[5] Bicket M.C., Chua K.P., Lagisetty P., Li Y., Waljee J.F., Brummett C.M., Nguyen T.D. (2024). Prevalence of Surgery Among Individuals in the United States. Ann. Surg. Open.

[6] O’Caoimh R., Sezgin D., O’Donovan M.R., Molloy D.W., Clegg A., Rockwood K., Liew A. (2021). Prevalence of frailty in 62 countries across the world: A systematic review and meta-analysis of population-level studies. Age Ageing.

[7] Sieber F., McIsaac D.I., Deiner S., Azefor T., Berger M., Hughes C., Leung J.M., Maldon J., McSwain J.R., Neuman M.D. (2025). 2025 American Society of Anesthesiologists Practice Advisory for Perioperative Care of Older Adults Scheduled for Inpatient Surgery. Anesthesiology.

[8] George E.L., Hall D.E., Youk A., Chen R., Kashikar A., Trickey A.W., Varley P.R., Shireman P.K., Shinall M.C., Massarweh N.N. (2021). Association Between Patient Frailty and Postoperative Mortality Across Multiple Noncardiac Surgical Specialties. JAMA Surg..

[9] Panayi A.C., Orkaby A.R., Sakthivel D., Endo Y., Varon D., Roh D., Orgill D.P., Neppl R.L., Javedan H., Bhasin S. (2019). Impact of frailty on outcomes in surgical patients: A systematic review and meta-analysis. Am. J. Surg..

[10] Kahlon S., Pederson J., Majumdar S.R., Belga S., Lau D., Fradette M., Boyko D., Bakal J.A., Johnston C., Padwal R.S. (2015). Association between frailty and 30-day outcomes after discharge from hospital. Can. Med. Assoc. J..

[11] Kojima G., Iliffe S., Jivraj S., Walters K. (2016). Association between frailty and quality of life among community-dwelling older people: A systematic review and meta-analysis. J. Epidemiol. Community Health.

[12] Kojima G. (2017). Frailty as a predictor of disabilities among community-dwelling older people: A systematic review and meta-analysis. Disabil. Rehabil..

[13] Joynt Maddox K.E., Elkind M.S.V., Aparicio H.J., Commodore-Mensah Y., de Ferranti S.D., Dowd W.N., Hernandez A.F., Khavjou O., Michos E.D., Palaniappan L. (2024). Forecasting the Burden of Cardiovascular Disease and Stroke in the United States Through 2050-Prevalence of Risk Factors and Disease: A Presidential Advisory from the American Heart Association. Circulation.

[14] Shi Q., Huang J., Wan J., Zhong Z., Sun Y., Zhou Y., Li J., Tan X., Yu B., Lu Y. (2024). Physical Frailty, Genetic Predisposition, and Incident Heart Failure. JACC Asia.

[15] Damluji A.A., Chung S.E., Xue Q.L., Hasan R.K., Moscucci M., Forman D.E., Bandeen-Roche K., Batchelor W., Walston J.D., Resar J.R. (2021). Frailty and cardiovascular outcomes in the National Health and Aging Trends Study. Eur. Heart J..

[16] McGinn R., Agung Y., Grudzinski A.L., Talarico R., Hallet J., McIsaac D.I. (2023). Attributable Perioperative Cost of Frailty after Major, Elective Noncardiac Surgery: A Population-based Cohort Study. Anesthesiology.

[17] Crooms R.C., Gelfman L.P. (2020). Palliative Care and End-of-Life Considerations for the Frail Patient. Anesth. Analg..

[18] Rockwood K., Mitnitski A. (2007). Frailty in relation to the accumulation of deficits. J. Gerontol. A Biol. Sci. Med. Sci..

[19] Fried L.P., Cohen A.A., Xue Q.L., Walston J., Bandeen-Roche K., Varadhan R. (2021). The physical frailty syndrome as a transition from homeostatic symphony to cacophony. Nat. Aging.

[20] Xue Q.L., Tian J., Walston J.D., Chaves P.H.M., Newman A.B., Bandeen-Roche K. (2020). Discrepancy in Frailty Identification: Move Beyond Predictive Validity. J. Gerontol. A Biol. Sci. Med. Sci..

[21] van Sleen Y., Shetty S.A., van der Heiden M., Venema M.C.A., Gutiérrez-Melo N., Toonen E.J.M., van Beek J., Buisman A., van Baarle D., Sauce D. (2023). Frailty is related to serum inflammageing markers: Results from the VITAL study. Immun. Ageing.

[22] Ferrucci L., Fabbri E. (2018). Inflammageing: Chronic inflammation in ageing, cardiovascular disease, and frailty. Nat. Rev. Cardiol..

[23] Roh J.S., Sohn D.H. (2018). Damage-Associated Molecular Patterns in Inflammatory Diseases. Immune Netw..

[24] Demaria M., Ohtani N., Youssef S.A., Rodier F., Toussaint W., Mitchell J.R., Laberge R.M., Vijg J., Van Steeg H., Dollé M.E. (2014). An essential role for senescent cells in optimal wound healing through secretion of PDGF-AA. Dev. Cell.

[25] Idda M.L., McClusky W.G., Lodde V., Munk R., Abdelmohsen K., Rossi M., Gorospe M. (2020). Survey of senescent cell markers with age in human tissues. Aging.

[26] Fielding R.A., Atkinson E.J., Aversa Z., White T.A., Heeren A.A., Achenbach S.J., Mielke M.M., Cummings S.R., Pahor M., Leeuwenburgh C. (2022). Associations between biomarkers of cellular senescence and physical function in humans: Observations from the lifestyle interventions for elders (LIFE) study. GeroScience.

[27] Picca A., Calvani R., Coelho-Júnior H.J., Marini F., Landi F., Marzetti E. (2022). Circulating Inflammatory, Mitochondrial Dysfunction, and Senescence-Related Markers in Older Adults with Physical Frailty and Sarcopenia: A BIOSPHERE Exploratory Study. Int. J. Mol. Sci..

[28] Bombelli S., Grasselli C., Mazzola P., Veronesi V., Morabito I., Zucchini N., Scollo C.M., Blanco S.I., De Marco S., Torsello B. (2024). Impairment of Renal and Hematopoietic Stem/Progenitor Cell Compartments in Frailty Syndrome: Link with Oxidative Stress, Plasma Cytokine Profiles, and Nuclear DNA Damage. J. Gerontol. A Biol. Sci. Med. Sci..

[29] Varadhan R., Chaves P.H., Lipsitz L.A., Stein P.K., Tian J., Windham B.G., Berger R.D., Fried L.P. (2009). Frailty and impaired cardiac autonomic control: New insights from principal components aggregation of traditional heart rate variability indices. J. Gerontol. A Biol. Sci. Med. Sci..

[30] Parvaneh S., Howe C.L., Toosizadeh N., Honarvar B., Slepian M.J., Fain M., Mohler J., Najafi B. (2015). Regulation of Cardiac Autonomic Nervous System Control across Frailty Statuses: A Systematic Review. Gerontology.

[31] Gielen E., Dupont J., Dejaeger M., Laurent M.R. (2023). Sarcopenia, osteoporosis and frailty. Metabolism.

[32] Aleixo G.F.P., Shachar S.S., Nyrop K.A., Muss H.B., Battaglini C.L., Williams G.R. (2020). Bioelectrical Impedance Analysis for the Assessment of Sarcopenia in Patients with Cancer: A Systematic Review. Oncologist.

[33] Buckinx F., Landi F., Cesari M., Fielding R.A., Visser M., Engelke K., Maggi S., Dennison E., Al-Daghri N.M., Allepaerts S. (2018). Pitfalls in the measurement of muscle mass: A need for a reference standard. J. Cachexia Sarcopenia Muscle.

[34] Cheng K.Y., Chow S.K., Hung V.W., Wong C.H., Wong R.M., Tsang C.S., Kwok T., Cheung W.H. (2021). Diagnosis of sarcopenia by evaluating skeletal muscle mass by adjusted bioimpedance analysis validated with dual-energy X-ray absorptiometry. J. Cachexia Sarcopenia Muscle.

[35] Cruz-Jentoft A.J., Sayer A.A. (2019). Sarcopenia. Lancet.

[36] Liu J., Chen X. (2023). Comparison between bioelectrical impedance analyses and dual-energy X-ray absorptiometry for accuracy in assessing appendicular skeletal muscle mass and diagnosing sarcopenia in hospitalized Chinese older adults. Medicine.

[37] Sousa-Santos A.R., Barros D., Montanha T.L., Carvalho J., Amaral T.F. (2021). Which is the best alternative to estimate muscle mass for sarcopenia diagnosis when DXA is unavailable?. Arch. Gerontol. Geriatr..

[38] Hanlon P., Wightman H., Politis M., Kirkpatrick S., Jones C., Andrew M.K., Vetrano D.L., Dent E., Hoogendijk E.O. (2024). The relationship between frailty and social vulnerability: A systematic review. Lancet Healthy Longev..

[39] Mitnitski A.B., Mogilner A.J., Rockwood K. (2001). Accumulation of deficits as a proxy measure of aging. Sci. World J..

[40] Kim D.J., Massa M.S., Potter C.M., Clarke R., Bennett D.A. (2022). Systematic review of the utility of the frailty index and frailty phenotype to predict all-cause mortality in older people. Syst. Rev..

[41] Fried L.P., Tangen C.M., Walston J., Newman A.B., Hirsch C., Gottdiener J., Seeman T., Tracy R., Kop W.J., Burke G. (2001). Frailty in older adults: Evidence for a phenotype. J. Gerontol. A Biol. Sci. Med. Sci..

[42] Ding Y.Y. (2017). Predictive Validity of Two Physical Frailty Phenotype Specifications Developed for Investigation of Frailty Pathways in Older People. Gerontology.

[43] Buta B.J., Walston J.D., Godino J.G., Park M., Kalyani R.R., Xue Q.L., Bandeen-Roche K., Varadhan R. (2016). Frailty assessment instruments: Systematic characterization of the uses and contexts of highly-cited instruments. Ageing Res. Rev..

[44] Rockwood K., Song X., MacKnight C., Bergman H., Hogan D.B., McDowell I., Mitnitski A. (2005). A global clinical measure of fitness and frailty in elderly people. Can. Med. Assoc. J..

[45] Amon J.N., Ridley E.J. (2022). Clinimetrics: Clinical Frailty Scale. J. Physiother..

[46] Rockwood K., Theou O. (2020). Using the Clinical Frailty Scale in Allocating Scarce Health Care Resources. Can. Geriatr. J..

[47] Savva G.M., Donoghue O.A., Horgan F., O’Regan C., Cronin H., Kenny R.A. (2013). Using timed up-and-go to identify frail members of the older population. J. Gerontol. A Biol. Sci. Med. Sci..

[48] Jung H.W., Kim S., Jang I.Y., Shin D.W., Lee J.E., Won C.W. (2020). Screening Value of Timed Up and Go Test for Frailty and Low Physical Performance in Korean Older Population: The Korean Frailty and Aging Cohort Study (KFACS). Ann. Geriatr. Med. Res..

[49] Sison S.D.M., Shi S.M., Kim K.M., Steinberg N., Jeong S., McCarthy E.P., Kim D.H. (2023). A crosswalk of commonly used frailty scales. J. Am. Geriatr. Soc..

[50] Ensrud K.E., Ewing S.K., Taylor B.C., Fink H.A., Cawthon P.M., Stone K.L., Hillier T.A., Cauley J.A., Hochberg M.C., Rodondi N. (2008). Comparison of 2 frailty indexes for prediction of falls, disability, fractures, and death in older women. Arch. Intern. Med..

[51] Morley J.E., Malmstrom T.K., Miller D.K. (2012). A simple frailty questionnaire (FRAIL) predicts outcomes in middle aged African Americans. J. Nutr. Health Aging.

[52] Saliba D., Elliott M., Rubenstein L.Z., Solomon D.H., Young R.T., Kamberg C.J., Roth C., MacLean C.H., Shekelle P.G., Sloss E.M. (2001). The Vulnerable Elders Survey: A tool for identifying vulnerable older people in the community. J. Am. Geriatr. Soc..

[53] Rolfson D.B., Majumdar S.R., Tsuyuki R.T., Tahir A., Rockwood K. (2006). Validity and reliability of the Edmonton Frail Scale. Age Ageing.

[54] Stevens P.E., Ahmed S.B., Carrero J.J., Foster B., Francis A., Hall R.K., Herrington W.G., Hill G., Inker L.A., Kazancıoğlu R. (2024). KDIGO 2024 Clinical Practice Guideline for the Evaluation and Management of Chronic Kidney Disease. Kidney Int..

[55] Fritzenschaft L., Boehm F., Rothenbacher D., Denkinger M., Dallmeier D. (2025). Association of blood biomarkers with frailty-A mapping review. Ageing Res. Rev..

[56] Li C., Ma Y., Yang C., Hua R., Xie W., Zhang L. (2022). Association of Cystatin C Kidney Function Measures with Long-term Deficit-Accumulation Frailty Trajectories and Physical Function Decline. JAMA Netw. Open.

[57] Potok O.A., Ix J.H., Shlipak M.G., Katz R., Hawfield A.T., Rocco M.V., Ambrosius W.T., Cho M.E., Pajewski N.M., Rastogi A. (2020). The Difference Between Cystatin C- and Creatinine-Based Estimated GFR and Associations with Frailty and Adverse Outcomes: A Cohort Analysis of the Systolic Blood Pressure Intervention Trial (SPRINT). Am. J. Kidney Dis..

[58] Potok O.A., Ix J.H., Shlipak M.G., Bansal N., Katz R., Kritchevsky S.B., Rifkin D.E. (2022). Cystatin C- and Creatinine-Based Glomerular Filtration Rate Estimation Differences and Muscle Quantity and Functional Status in Older Adults: The Health, Aging, and Body Composition Study. Kidney Med..

[59] Yuan J.H., Rifkin D.E., Ginsberg C., Cawthon P.M., Kado D.M., Bauer S.R., Ensrud K.E., Hoffman A.R., Potok O.A. (2024). Difference between kidney function by cystatin C versus creatinine and association with muscle mass and frailty. J. Am. Geriatr. Soc..

[60] Yao S., Guo J., Shi G., Zhu Y., Wang Y., Chu X., Jiang X., Jin L., Wang Z., Wang X. (2019). Association of BNP with Frailty in Elderly Population: Rugao Longevity and Ageing Study. J. Nutr. Health Aging.

[61] Camino-Willhuber G., Tani S., Schonnagel L., Caffard T., Haffer H., Chiapparelli E., Sarin M., Shue J., Soffin E.M., Zelenty W.D. (2023). Association of Frailty and Preoperative Hypoalbuminemia with the Risk of Complications, Readmission, and Mortality After Spine Surgery. World Neurosurg..

[62] Zeng P., Li M., Cao J., Zeng L., Jiang C., Lin F. (2024). Association of metabolic syndrome severity with frailty progression among Chinese middle and old-aged adults: A longitudinal study. Cardiovasc. Diabetol..

[63] Rattray N.J.W., Trivedi D.K., Xu Y., Chandola T., Johnson C.H., Marshall A.D., Mekli K., Rattray Z., Tampubolon G., Vanhoutte B. (2019). Metabolic dysregulation in vitamin E and carnitine shuttle energy mechanisms associate with human frailty. Nat. Commun..

[64] Westbrook R., Zhang C., Yang H., Tian J., Guo S., Xue Q.L., Walston J., Le A., Abadir P.M. (2022). Metabolomics-Based Identification of Metabolic Dysfunction in Frailty. J. Gerontol. A Biol. Sci. Med. Sci..

[65] Calvani R., Picca A., Rodriguez-Manas L., Tosato M., Coelho-Junior H.J., Biancolillo A., Laosa O., Gervasoni J., Primiano A., Santucci L. (2023). Amino Acid Profiles in Older Adults with Frailty: Secondary Analysis from MetaboFrail and BIOSPHERE Studies. Metabolites.

[66] Ni Lochlainn M., Cox N.J., Wilson T., Hayhoe R.P.G., Ramsay S.E., Granic A., Isanejad M., Roberts H.C., Wilson D., Welch C. (2021). Nutrition and Frailty: Opportunities for Prevention and Treatment. Nutrients.

[67] Ye Y., Noche R.B., Szejko N., Both C.P., Acosta J.N., Leasure A.C., Brown S.C., Sheth K.N., Gill T.M., Zhao H. (2023). A genome-wide association study of frailty identifies significant genetic correlation with neuropsychiatric, cardiovascular, and inflammation pathways. GeroScience.

[68] Yáñez-Mó M., Siljander P.R., Andreu Z., Zavec A.B., Borràs F.E., Buzas E.I., Buzas K., Casal E., Cappello F., Carvalho J. (2015). Biological properties of extracellular vesicles and their physiological functions. J. Extracell. Vesicles.

[69] Ipson B.R., Fletcher M.B., Espinoza S.E., Fisher A.L. (2018). Identifying Exosome-Derived MicroRNAs as Candidate Biomarkers of Frailty. J. Frailty Aging.

[70] Machida T., Tomofuji T., Ekuni D., Maruyama T., Yoneda T., Kawabata Y., Mizuno H., Miyai H., Kunitomo M., Morita M. (2015). MicroRNAs in Salivary Exosome as Potential Biomarkers of Aging. Int. J. Mol. Sci..

[71] Wang B., Yin Z., Lin Y., Deng X., Liu F., Tao H., Dong R., Lin X., Bi Y. (2022). Correlation between microRNA-320 and postoperative delirium in patients undergoing tibial fracture internal fixation surgery. BMC Anesthesiol..

[72] Kern A.E., Pereyra D., Santol J., Ammann M., Kim S., Huber F.X., Weninger J., Brunner S., Laferl V., Herrmann Y. (2025). MicroRNA based Prediction of Posthepatectomy Liver Failure and Mortality Outperforms Established Markers of Preoperative Risk Assessment. Ann. Surg. Oncol..

[73] Rusanova I., Fernández-Martínez J., Fernández-Ortiz M., Aranda-Martínez P., Escames G., García-García F.J., Mañas L., Acuña-Castroviejo D. (2019). Involvement of plasma miRNAs, muscle miRNAs and mitochondrial miRNAs in the pathophysiology of frailty. Exp. Gerontol..

[74] Rusanova I., Diaz-Casado M.E., Fernández-Ortiz M., Aranda-Martínez P., Guerra-Librero A., García-García F.J., Escames G., Mañas L., Acuña-Castroviejo D. (2018). Analysis of Plasma MicroRNAs as Predictors and Biomarkers of Aging and Frailty in Humans. Oxidative Med. Cell. Longev..

[75] Olivieri F., Spazzafumo L., Santini G., Lazzarini R., Albertini M.C., Rippo M.R., Galeazzi R., Abbatecola A.M., Marcheselli F., Monti D. (2012). Age-related differences in the expression of circulating microRNAs: miR-21 as a new circulating marker of inflammaging. Mech. Ageing Dev..

[76] Mak J.K.L., Skovgaard A.C., Nygaard M., Kananen L., Reynolds C.A., Wang Y., Kuja-Halkola R., Karlsson I.K., Pedersen N.L., Hägg S. (2024). Epigenome-wide analysis of frailty: Results from two European twin cohorts. Aging Cell.

[77] Atkins J.L., Jylhävä J., Pedersen N.L., Magnusson P.K., Lu Y., Wang Y., Hägg S., Melzer D., Williams D.M., Pilling L.C. (2021). A genome-wide association study of the frailty index highlights brain pathways in ageing. Aging Cell.

[78] Vatic M., von Haehling S., Ebner N. (2020). Inflammatory biomarkers of frailty. Exp. Gerontol..

[79] Xu Y., Wang M., Chen D., Jiang X., Xiong Z. (2022). Inflammatory biomarkers in older adults with frailty: A systematic review and meta-analysis of cross-sectional studies. Aging Clin. Exp. Res..

[80] Kamper R.S., Alcazar J., Andersen L.L., Haddock B., Jorgensen N.R., Hovind P., Suetta C. (2021). Associations between inflammatory markers, body composition, and physical function: The Copenhagen Sarcopenia Study. J. Cachexia Sarcopenia Muscle.

[81] Mekli K., Nazroo J.Y., Marshall A.D., Kumari M., Pendleton N. (2016). Proinflammatory genotype is associated with the frailty phenotype in the English Longitudinal Study of Ageing. Aging Clin. Exp. Res..

[82] Collerton J., Martin-Ruiz C., Davies K., Hilkens C.M., Isaacs J., Kolenda C., Parker C., Dunn M., Catt M., Jagger C. (2012). Frailty and the role of inflammation, immunosenescence and cellular ageing in the very old: Cross-sectional findings from the Newcastle 85+ Study. Mech. Ageing Dev..

[83] Langmann G.A., Perera S., Ferchak M.A., Nace D.A., Resnick N.M., Greenspan S.L. (2017). Inflammatory Markers and Frailty in Long-Term Care Residents. J. Am. Geriatr. Soc..

[84] Puts M.T., Visser M., Twisk J.W., Deeg D.J., Lips P. (2005). Endocrine and inflammatory markers as predictors of frailty. Clin. Endocrinol..

[85] Luo Y.F., Cheng Z.J., Wang Y.F., Jiang X.Y., Lei S.F., Deng F.Y., Ren W.Y., Wu L.F. (2024). Unraveling the relationship between high-sensitivity C-reactive protein and frailty: Evidence from longitudinal cohort study and genetic analysis. BMC Geriatr..

[86] Walker K.A., Walston J., Gottesman R.F., Kucharska-Newton A., Palta P., Windham B.G. (2019). Midlife Systemic Inflammation Is Associated with Frailty in Later Life: The ARIC Study. J. Gerontol. A Biol. Sci. Med. Sci..

[87] Wang D., Day E.A., Townsend L.K., Djordjevic D., Jørgensen S.B., Steinberg G.R. (2021). GDF15: Emerging biology and therapeutic applications for obesity and cardiometabolic disease. Nat. Rev. Endocrinol..

[88] Conte M., Martucci M., Mosconi G., Chiariello A., Cappuccilli M., Totti V., Santoro A., Franceschi C., Salvioli S. (2020). GDF15 Plasma Level Is Inversely Associated with Level of Physical Activity and Correlates with Markers of Inflammation and Muscle Weakness. Front. Immunol..

[89] Kamper R.S., Nygaard H., Praeger-Jahnsen L., Ekmann A., Ditlev S.B., Schultz M., Hansen S.K., Hansen P., Pressel E., Suetta C. (2024). GDF-15 is associated with sarcopenia and frailty in acutely admitted older medical patients. J. Cachexia Sarcopenia Muscle.

[90] Kim M., Walston J.D., Won C.W. (2022). Associations Between Elevated Growth Differentiation Factor-15 and Sarcopenia Among Community-dwelling Older Adults. J. Gerontol. A Biol. Sci. Med. Sci..

[91] Álvarez-Satta M., Berna-Erro A., Carrasco-Garcia E., Alberro A., Saenz-Antoñanzas A., Vergara I., Otaegui D., Matheu A. (2020). Relevance of oxidative stress and inflammation in frailty based on human studies and mouse models. Aging.

[92] Liu C.K., Lyass A., Larson M.G., Massaro J.M., Wang N., D’Agostino R.B., Benjamin E.J., Murabito J.M. (2016). Biomarkers of oxidative stress are associated with frailty: The Framingham Offspring Study. Age.

[93] Inglés M., Gambini J., Carnicero J.A., García-García F.J., Rodríguez-Mañas L., Olaso-González G., Dromant M., Borrás C., Viña J. (2014). Oxidative stress is related to frailty, not to age or sex, in a geriatric population: Lipid and protein oxidation as biomarkers of frailty. J. Am. Geriatr. Soc..

[94] Das A., Cumming R.G., Naganathan V., Blyth F., Ribeiro R.V., Le Couteur D.G., Handelsman D.J., Waite L.M., Simpson S.J., Hirani V. (2020). Prospective Associations Between Dietary Antioxidant Intake and Frailty in Older Australian Men: The Concord Health and Ageing in Men Project. J. Gerontol. A Biol. Sci. Med. Sci..

[95] Ble A., Cherubini A., Volpato S., Bartali B., Walston J.D., Windham B.G., Bandinelli S., Lauretani F., Guralnik J.M., Ferrucci L. (2006). Lower plasma vitamin E levels are associated with the frailty syndrome: The InCHIANTI study. J. Gerontol. A Biol. Sci. Med. Sci..

[96] Saum K.U., Dieffenbach A.K., Jansen E.H., Schöttker B., Holleczek B., Hauer K., Brenner H. (2015). Association between Oxidative Stress and Frailty in an Elderly German Population: Results from the ESTHER Cohort Study. Gerontology.

[97] Sajeev S., Champion S., Maeder A., Gordon S. (2022). Machine learning models for identifying pre-frailty in community dwelling older adults. BMC Geriatr..

[98] Tseng W.H., Chattopadhyay A., Phan N.N., Chuang E.Y., Lee O.K. (2024). Utilizing multimodal approach to identify candidate pathways and biomarkers and predicting frailty syndrome in individuals from UK Biobank. GeroScience.

[99] Conte M., Sevini F., Conte G., Tognocchi M., Ciurca E., Trofarello L., Chiariello A., Capri M., Franceschi C., Monti D. (2025). The combination of GDF15, FGF21, sRAGE and NfL plasma levels can identify frailty in community-dwelling people across old age. Mech. Ageing Dev..

[100] Chou Y.Y., Wang M.S., Lin C.F., Lee Y.S., Lee P.H., Huang S.M., Wu C.L., Lin S.Y. (2024). The application of machine learning for identifying frailty in older patients during hospital admission. BMC Med. Inform. Decis. Mak..

[101] Cruz-Jentoft A.J., Bahat G., Bauer J., Boirie Y., Bruyère O., Cederholm T., Cooper C., Landi F., Rolland Y., Sayer A.A. (2019). Sarcopenia: Revised European consensus on definition and diagnosis. Age Ageing.

[102] Guglielmi G., Ponti F., Agostini M., Amadori M., Battista G., Bazzocchi A. (2016). The role of DXA in sarcopenia. Aging Clin. Exp. Res..

[103] Derstine B.A., Holcombe S.A., Ross B.E., Wang N.C., Su G.L., Wang S.C. (2021). Optimal body size adjustment of L3 CT skeletal muscle area for sarcopenia assessment. Sci. Rep..

[104] Goodpaster B.H., Carlson C.L., Visser M., Kelley D.E., Scherzinger A., Harris T.B., Stamm E., Newman A.B. (2001). Attenuation of skeletal muscle and strength in the elderly: The Health ABC Study. J. Appl. Physiol..

[105] Beavers K.M., Beavers D.P., Houston D.K., Harris T.B., Hue T.F., Koster A., Newman A.B., Simonsick E.M., Studenski S.A., Nicklas B.J. (2013). Associations between body composition and gait-speed decline: Results from the Health, Aging, and Body Composition study. Am. J. Clin. Nutr..

[106] Thai S.T., Lund J.L., Poole C., Buse J.B., Stürmer T., Harmon C.A., Al-Obaidi M., Williams G.R. (2024). Skeletal muscle density performance for screening frailty in older adults with cancer and the impact of diabetes: The CARE Registry. J. Geriatr. Oncol..

[107] Wang L., Yin L., Zhao Y., Su Y., Sun W., Chen S., Liu Y., Yang M., Yu A., Guglielmi G. (2021). Muscle Density, but Not Size, Correlates Well with Muscle Strength and Physical Performance. J. Am. Med. Dir. Assoc..

[108] Santanasto A.J., Zmuda J.M., Cvejkus R.K., Gordon C.L., Nair S., Carr J.J., Terry J.G., Wheeler V.W., Miljkovic I. (2023). Thigh and Calf Myosteatosis are Strongly Associated with Muscle and Physical Function in African Caribbean Men. J. Gerontol. A Biol. Sci. Med. Sci..

[109] Lenchik L., Lenoir K.M., Tan J., Boutin R.D., Callahan K.E., Kritchevsky S.B., Wells B.J. (2019). Opportunistic Measurement of Skeletal Muscle Size and Muscle Attenuation on Computed Tomography Predicts 1-Year Mortality in Medicare Patients. J. Gerontol. A Biol. Sci. Med. Sci..

[110] Reinders I., Murphy R.A., Brouwer I.A., Visser M., Launer L., Siggeirsdottir K., Eiriksdottir G., Gudnason V., Jonsson P.V., Lang T.F. (2016). Muscle Quality and Myosteatosis: Novel Associations with Mortality Risk: The Age, Gene/Environment Susceptibility (AGES)-Reykjavik Study. Am. J. Epidemiol..

[111] Nachit M., Horsmans Y., Summers R.M., Leclercq I.A., Pickhardt P.J. (2023). AI-based CT Body Composition Identifies Myosteatosis as Key Mortality Predictor in Asymptomatic Adults. Radiology.

[112] Cheung A., Haas B., Ringer T.J., McFarlan A., Wong C.L. (2017). Canadian Study of Health and Aging Clinical Frailty Scale: Does It Predict Adverse Outcomes among Geriatric Trauma Patients?. J. Am. Coll. Surg..

[113] Di Cola S., D’Amico G., Caraceni P., Schepis F., Loredana S., Lampertico P., Toniutto P., Martini S., Maimone S., Colecchia A. (2024). Myosteatosis is closely associated with sarcopenia and significantly worse outcomes in patients with cirrhosis. J. Hepatol..

[114] Ebadi M., Tsien C., Bhanji R.A., Dunichand-Hoedl A.R., Rider E., Motamedrad M., Mazurak V.C., Baracos V., Montano-Loza A.J. (2022). Skeletal Muscle Pathological Fat Infiltration (Myosteatosis) Is Associated with Higher Mortality in Patients with Cirrhosis. Cells.

[115] Erlandson K.M., Umbleja T., Lu M.T., Taron J., Ribaudo H.J., Overton E.T., Presti R.M., Haas D.W., Sax P.E., Yin M.T. (2023). Associations of Muscle Density and Area with Coronary Artery Plaque and Physical Function. J. Acquir. Immune Defic. Syndr..

[116] Horwich T.B., Fonarow G.C., Clark A.L. (2018). Obesity and the Obesity Paradox in Heart Failure. Prog. Cardiovasc. Dis..

[117] Gravina G., Ferrari F., Nebbiai G. (2021). The obesity paradox and diabetes. Eat. Weight. Disord.-Stud. Anorex. Bulim. Obes..

[118] Yao S., Zeng L., Wang F., Chen K. (2023). Obesity Paradox in Lung Diseases: What Explains It?. Obes. Facts.

[119] Gao Q., Mei F., Shang Y., Hu K., Chen F., Zhao L., Ma B. (2021). Global prevalence of sarcopenic obesity in older adults: A systematic review and meta-analysis. Clin. Nutr..

[120] Gao Q., Hu K., Gao J., Shang Y., Mei F., Zhao L., Chen F., Ma B. (2022). Prevalence and prognostic value of sarcopenic obesity in patients with cancer: A systematic review and meta-analysis. Nutrition.

[121] Batsis J.A., Mackenzie T.A., Barre L.K., Lopez-Jimenez F., Bartels S.J. (2014). Sarcopenia, sarcopenic obesity and mortality in older adults: Results from the National Health and Nutrition Examination Survey III. Eur. J. Clin. Nutr..

[122] Chan W., Chin S.H., Whittaker A.C., Jones D., Kaur O., Bosch J.A., Borrows R. (2019). The Associations of Muscle Strength, Muscle Mass, and Adiposity with Clinical Outcomes and Quality of Life in Prevalent Kidney Transplant Recipients. J. Ren. Nutr..

[123] Chauvot de Beauchene R., Souweine B., Bonnet B., Evrard B., Boirie Y., Cassagnes L., Dupuis C. (2025). Sarcopenia, myosteatosis and inflammation are independent prognostic factors of SARS-CoV-2 pneumonia patients admitted to the ICU. Sci. Rep..

[124] Bonatti M., Lombardo F., Valletta R., Comai A., Petralia B., Avesani G., Franchini E., Rossi A., De Santis N., Guerriero M. (2023). Myosteatosis as an independent predictor of short-term mortality in successfully reperfused acute ischemic stroke. Neuroradiol. J..

[125] Park B., Vandal A., Welsh F., Eglinton T., Koea J., Taneja A., Barazanchi A., Hill A.G., MacCormick A.D. (2025). Sarcopenia, myosteatosis, and frailty parameters to predict adverse outcomes in patients undergoing emergency laparotomy: Prospective observational multicentre cohort study. BJS Open.

[126] Fumagalli I.A., Le S.T., Peng P.D., Kipnis P., Liu V.X., Caan B., Chow V., Beg M.F., Popuri K., Cespedes Feliciano E.M. (2024). Automated CT Analysis of Body Composition as a Frailty Biomarker in Abdominal Surgery. JAMA Surg..

[127] Tokuda T., Yamamoto M., Kagase A., Koyama Y., Otsuka T., Tada N., Naganuma T., Araki M., Yamanaka F., Shirai S. (2020). Importance of combined assessment of skeletal muscle mass and density by computed tomography in predicting clinical outcomes after transcatheter aortic valve replacement. Int. J. Cardiovasc. Imaging.

[128] Soud M., Alahdab F., Ho G., Kuku K.O., Cejudo-Tejeda M., Hideo-Kajita A., de Araujo Gonçalves P., Teles R.C., Waksman R., Garcia-Garcia H.M. (2019). Usefulness of skeletal muscle area detected by computed tomography to predict mortality in patients undergoing transcatheter aortic valve replacement: A meta-analysis study. Int. J. Cardiovasc. Imaging.

[129] Huangfu G., Kumar A.A., Yong G., Shetty S., He A., Raju V., Dwivedi G., Ihdayhid A.R. (2024). CT-derived frailty score outperforms clinical frailty scale for mortality prediction following TAVR. J. Cardiovasc. Comput. Tomogr..

[130] Bradley N.A., Walter A., Roxburgh C.S.D., McMillan D.C., Guthrie G.J.K. (2024). The Relationship between Clinical Frailty Score, CT-Derived Body Composition, Systemic Inflammation, and Survival in Patients with Chronic Limb-Threatening Ischemia. Ann. Vasc. Surg..

[131] Kärkkäinen J.M., Tenorio E.R., Oksala N., Macedo T.A., Sen I., Mendes B.C., DeMartino R.R., Jacobs M.J., Mees B., Oderich G.S. (2020). Pre-operative Psoas Muscle Size Combined with Radiodensity Predicts Mid-Term Survival and Quality of Life After Fenestrated-Branched Endovascular Aortic Repair. Eur. J. Vasc. Endovasc. Surg..

[132] Kärkkäinen J.M., Oderich G.S., Tenorio E.R., Pather K., Oksala N., Macedo T.A., Vrtiska T., Mees B., Jacobs M.J. (2021). Psoas muscle area and attenuation are highly predictive of complications and mortality after complex endovascular aortic repair. J. Vasc. Surg..

[133] Morel A., Ouamri Y., Canouï-Poitrine F., Mulé S., Champy C.M., Ingels A., Audard V., Luciani A., Grimbert P., Matignon M. (2022). Myosteatosis as an independent risk factor for mortality after kidney allograft transplantation: A retrospective cohort study. J. Cachexia Sarcopenia Muscle.

[134] Xiao J., Caan B.J., Cespedes Feliciano E.M., Meyerhardt J.A., Peng P.D., Baracos V.E., Lee V.S., Ely S., Gologorsky R.C., Weltzien E. (2020). Association of Low Muscle Mass and Low Muscle Radiodensity with Morbidity and Mortality for Colon Cancer Surgery. JAMA Surg..

[135] Margadant C.C., Bruns E.R., Sloothaak D.A., van Duijvendijk P., van Raamt A.F., van der Zaag H.J., Buskens C.J., van Munster B.C., van der Zaag E.S. (2016). Lower muscle density is associated with major postoperative complications in older patients after surgery for colorectal cancer. Eur. J. Surg. Oncol..

[136] Kroenke C.H., Prado C.M., Meyerhardt J.A., Weltzien E.K., Xiao J., Cespedes Feliciano E.M., Caan B.J. (2018). Muscle radiodensity and mortality in patients with colorectal cancer. Cancer.

[137] Murnane L.C., Forsyth A.K., Koukounaras J., Pilgrim C.H., Shaw K., Brown W.A., Mourtzakis M., Tierney A.C., Burton P.R. (2021). Myosteatosis predicts higher complications and reduced overall survival following radical oesophageal and gastric cancer surgery. Eur. J. Surg. Oncol..

[138] Haehl E., Alvino L., Rühle A., Zou J., Fabian A., Grosu A.L., Nicolay N.H. (2022). Sarcopenia as a Prognostic Marker in Elderly Head and Neck Squamous Cell Carcinoma Patients Undergoing (Chemo-)Radiation. Cancers.

[139] Canales C., Mazor E., Coy H., Grogan T.R., Duval V., Raman S., Cannesson M., Singh S.P. (2022). Preoperative Point-of-Care Ultrasound to Identify Frailty and Predict Postoperative Outcomes: A Diagnostic Accuracy Study. Anesthesiology.

[140] Jiang R., Noble S., Sui J., Yoo K., Rosenblatt M., Horien C., Qi S., Liang Q., Sun H., Calhoun V.D. (2023). Associations of physical frailty with health outcomes and brain structure in 483 033 middle-aged and older adults: A population-based study from the UK Biobank. Lancet Digit. Health.

[141] Nishita Y., Nakamura A., Kato T., Otsuka R., Iwata K., Tange C., Ando F., Ito K., Shimokata H., Arai H. (2019). Links Between Physical Frailty and Regional Gray Matter Volumes in Older Adults: A Voxel-Based Morphometry Study. J. Am. Med. Dir. Assoc..

[142] Apóstolo J., Cooke R., Bobrowicz-Campos E., Santana S., Marcucci M., Cano A., Vollenbroek-Hutten M., Germini F., D’Avanzo B., Gwyther H. (2018). Effectiveness of interventions to prevent pre-frailty and frailty progression in older adults: A systematic review. JBI Evid. Synth..

[143] Travers J., Romero-Ortuno R., Langan J., MacNamara F., McCormack D., McDermott C., McEntire J., McKiernan J., Lacey S., Doran P. (2023). Building resilience and reversing frailty: A randomised controlled trial of a primary care intervention for older adults. Age Ageing.

[144] An J., Ryu H.K., Lyu S.J., Yi H.J., Lee B.H. (2021). Effects of Preoperative Telerehabilitation on Muscle Strength, Range of Motion, and Functional Outcomes in Candidates for Total Knee Arthroplasty: A Single-Blind Randomized Controlled Trial. Int. J. Environ. Res. Public Health.

[145] Lai Y., Huang J., Yang M., Su J., Liu J., Che G. (2017). Seven-day intensive preoperative rehabilitation for elderly patients with lung cancer: A randomized controlled trial. J. Surg. Res..

[146] Carli F., Bousquet-Dion G., Awasthi R., Elsherbini N., Liberman S., Boutros M., Stein B., Charlebois P., Ghitulescu G., Morin N. (2020). Effect of Multimodal Prehabilitation vs Postoperative Rehabilitation on 30-Day Postoperative Complications for Frail Patients Undergoing Resection of Colorectal Cancer: A Randomized Clinical Trial. JAMA Surg..

[147] Molenaar C.J.L., Minnella E.M., Coca-Martinez M., Ten Cate D.W.G., Regis M., Awasthi R., Martinez-Palli G., Lopez-Baamonde M., Sebio-Garcia R., Feo C.V. (2023). Effect of Multimodal Prehabilitation on Reducing Postoperative Complications and Enhancing Functional Capacity Following Colorectal Cancer Surgery: The PREHAB Randomized Clinical Trial. JAMA Surg..

